# Spatiotemporally Precise Optical Manipulation of Intracellular Molecular Activities

**DOI:** 10.1002/advs.202307342

**Published:** 2024-01-26

**Authors:** Bin Dong, Shivam Mahapatra, Matthew G. Clark, Mark S. Carlsen, Karsten J. Mohn, Seohee Ma, Kent A. Brasseale, Grace Crim, Chi Zhang

**Affiliations:** ^1^ Department of Chemistry Purdue University 560 Oval Dr. West Lafayette IN 47907 USA; ^2^ Purdue Center for Cancer Research 201 S. University St. West Lafayette IN 47907 USA; ^3^ Purdue Institute of Inflammation, Immunology, and Infectious Disease 207 S. Martin Jischke Dr. West Lafayette IN 47907 USA

**Keywords:** confocal fluorescence microscopy, fluorescent proteins, optical manipulation, optical treatment, organelle activities

## Abstract

Controlling chemical processes in live cells is a challenging task. The spatial heterogeneity of biochemical reactions in cells is often overlooked by conventional means of incubating cells with desired chemicals. A comprehensive understanding of spatially diverse biochemical processes requires precise control over molecular activities at the subcellular level. Herein, a closed‐loop optoelectronic control system is developed that allows the manipulation of biomolecular activities in live cells at high spatiotemporal precision. Chemical‐selective fluorescence signals are utilized to command lasers that trigger specific chemical processes or control the activation of photoswitchable inhibitors at desired targets. This technology is fully compatible with laser scanning confocal fluorescence microscopes. The authors demonstrate selective interactions of a 405 nm laser with targeted organelles and simultaneous monitoring of cell responses by fluorescent protein signals. Notably, blue laser interaction with the endoplasmic reticulum leads to a more pronounced reduction in cytosolic green fluorescent protein signals in comparison to that with nuclei and lipid droplets. Moreover, when combined with a photoswitchable inhibitor, microtubule polymerization is selectively inhibited within the subcellular compartments. This technology enables subcellular spatiotemporal optical manipulation over chemical processes and drug activities, exclusively at desired targets, while minimizing undesired effects on non‐targeted locations.

## Graphical abstract

A continuous wave real‐time precision opto‐control (CW‐RPOC) system is developed. This technology provides spatially precise subcellular control over chemical processes and drug activities, minimizing undesired effects at non‐target sites. Applying CW‐RPOC, the selective generation of reactive oxygen species is demonstrated solely at desired organelles and the inhibition of microtubule polymerization is achieved at subcellular locations.

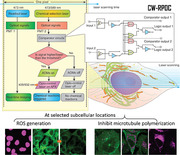

## 1 Introduction

Biomolecular activities within cells exhibit intricate spatiotemporal heterogeneity. Advanced microscopy technologies have provided the means to visualize and study this heterogeneity in real time and at high spatial resolution.^[^ ^1^, ^2^, ^3^, ^4^, ^5^ ^]^ However, current methods of controlling chemical processes in live cells remain rudimentary and often fail to address this heterogeneity. The prevailing approach involves incubating cells with chemical compounds that interact with molecular entities. However, these interactions lack spatiotemporal precision, leading to potential off‐target effects. The ability to precisely manipulate protein or drug activities in space and time would provide valuable insights into biological functions and drug‐target interactions. Laser‐based optical control technologies offer the potential to achieve high spatiotemporal precision.^[^ ^6^, ^7^, ^8^, ^9^ ^]^ Laser beams can be focused to hundreds of nanometers and delivered instantaneously to targets of interest. Existing optical control methods rely on microscopes to gather prior knowledge of the sample and visualize sample responses.^[^ ^10^, ^11^, ^12^ ^]^ After image acquisition and analysis, laser interaction sites are manually selected using galvo mirrors or structured illumination methods.^[^ ^8^, ^10^, ^11^, ^12^ ^]^ These requirements do not allow real‐time precise selection and optical manipulation of dynamic proteins or organelles in cells.

To address these challenges, we introduced the concept of real‐time precision opto‐control (RPOC) which is based on a closed‐loop optoelectronic feedback system paired with a laser scanning microscope.^[^ ^13^ ^]^ This technology can detect chemical‐specific optical signals in live cells, decide pixels of interaction in real time, and manipulate chemical processes with high spatiotemporal precision solely at targets of interest. However, the prototype of RPOC is built upon a costly femtosecond laser source and has poor sensitivity for the readout of cellular responses.^[^ ^13^ ^]^ Moreover, it only allows to command a single laser for optical manipulation. Here, we reinvented RPOC based on a confocal fluorescence microscope and advanced optoelectronic feedback. The use of four continuous wave (CW) lasers greatly reduces the system cost and enables simultaneous target selection, optical control, and monitoring of cellular responses. A new comparator circuitry and optoelectronic feedback system can actively determine and separately command multiple laser wavelengths at desired pixels within the pixel dwell time. Active pixels (APXs) are automatically selected based on the chemical signatures, eliminating the need for a priori knowledge of the sample. Using the CW‐RPOC technology, we demonstrated the selective perturbation of distinct organelles using a 405 nm laser and quantified the cellular responses using fluorescent protein signals. Quantitative analysis and laser dosage dependence results indicate that treating the endoplasmic reticulum (ER), compared to lipid droplets (LDs) and nuclei, leads to a more pronounced loss of cytosolic fluorescent protein signals. Furthermore, by coupling a photoswitchable inhibitor with RPOC, we achieve selective inhibition of tubulin polymerization in subcellular compartments. Being able to control separate laser wavelengths simultaneously enables the activation of inhibitors exclusively at desired targets while inactivating the same molecules at unwanted locations. This prevents activated inhibitors from interacting with undesired areas through diffusion. CW‐RPOC is fully compatible with confocal fluorescence microscopes and extends the imaging system for highly precise optical manipulation and simultaneously sensitive quantitation of cell responses.

## 2 Results

### 2.1 CW‐RPOC for Spatiotemporal Manipulation of Biomolecules

**Figure 1:**
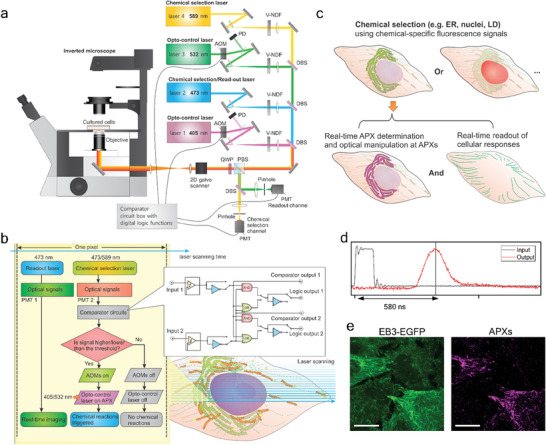
The CW‐RPOC platform for precision opto‐control. a) The optical configuration of the CW‐RPOC system. AOM, acousto‐optic modulator; DBS, dichroic beamsplitter; PBS, polarizing beamsplitter; V‐NDF, variable neutral density filter; QWP, quarter‐wave plate; PMT, photomultiplier tube. b) The workflow of CW‐RPOC at a single pixel and the configuration of the comparator circuitry. APX, active pixel. c) An illustration of selective chemical detection, APX determination, and real‐time monitoring of cell responses using the CW‐RPOC system. d) The measurement of the response time of the RPOC system for the 405 nm laser. e) A fluorescence image of EB3‐EGFP signals from HeLa cells and the corresponding APXs selected using the fluorescence signals. Scale bars are 10 µm.

The CW‐RPOC system is based on a laser‐scanning confocal fluorescence microscope that enables organelle detection, imaging, and spatially precise optical manipulation. As illustrated in **Figure** **1**a, four CW lasers facilitate signal excitation and optical treatment. The system encompasses three key components: Optical target selection, optical manipulation, and readout of cell responses. Both the 473 and 589 nm lasers are utilized to excite fluorescent labels such as ER‐Tracker, BODIPY LD stain, and mCherry‐histone‐2B (H2B) conjugation and so on, to identify targets of interest. The 473 nm laser, which matches the excitation wavelength of enhanced green fluorescent proteins (EGFPs) that are sensitive to cellular perturbations, also functions as the laser for the readout of cell responses. The 405 and 532 nm lasers are used as action lasers to treat or manipulate the desired entities. A 405 nm laser is directly used to perturb desired organelles, and very low power of both 405 and 532 nm lasers is used to control photoswitchable inhibitors. Two photomultiplier tube (PMT) detectors are set up in confocal configurations. One PMT is utilized to detect target signals that trigger optical manipulation, while the other is designated as a readout detector for measuring cellular EGFP responses. Fluorescence signals from labeled organelles are first collected by the PMT and then sent to a comparator circuit for decision‐making. This circuit is responsible for comparing the fluorescent signals with preset thresholds and triggering acousto‐optic modulators (AOMs) based on the result of the comparison. The preset threshold can be tuned by the knobs on the box or set by an analog/digital input. If the fluorescence signal intensity exceeds the preset threshold, a high transistor‐transistor logic (TTL) serial command is sent to the AOM to diffract the optical manipulation laser. The first‐order diffraction beam is combined with other laser beams and directed into the microscope. Otherwise, a low TTL serial command is generated, which does not trigger the AOM diffraction, preventing the coupling of the manipulation laser into the microscope. A detailed illustration of the two‐channel comparator circuit is discussed in the Supporting Information (Figure S1, and Note S1, Supporting Information). The AOMs in the 405 and the 532 nm laser paths are controlled separately based on the optical signal and the preset threshold at each pixel (Figures S2 and S3, Supporting Information). The flow of the CW‐RPOC is shown in Figure 1b. The use of CW lasers significantly reduces the cost and complexity of the system compared to its predecessor (Note S2, Supporting Information).^[^ ^13^ ^]^ The optical target selection, decision‐making, and optical manipulation are performed within a single pixel, paired with simultaneous real‐time imaging of cells from the readout channel. An APX is defined as the pixel on which the optical manipulation laser is activated.^[^ ^13^ ^]^ APXs can be visualized by using photodiodes collecting small fractions of the diffracted optical manipulation lasers or a copy of the comparator circuit TTL output. The APX intensity quantifies the dosage of the treatment laser at each pixel.

APXs can be chosen exclusively for desired targets in cells. For example, as illustrated in Figure 1c, fluorescent labels can be used to select APXs solely on ER, nuclei, LD, etc. Tuning the intensity threshold on the comparator circuit box, APXs can be selected on the organelles to overlap with the fluorescent signals of these organelles to different extents (0% to 100%). The readout signal from EGFP allows simultaneous monitoring of cellular responses to the organelle‐specific laser treatment. The treatment laser power, treatment time, and wavelength can be separately controlled to explore the impact of laser dosage and wavelength on cellular activities. The response time of the feedback loop, as measured to be ≈580 ns for the 405 nm laser (Figure 1d, and Figure S4 and Note S3, Supporting Information), is significantly shorter than the 10–20 µs pixel dwell time. This rapid response time ensures tracking and real‐time control of dynamic molecular entities in cells. Figure 1e displays fluorescence signals from EGFP conjugated with end‐binding protein 3 (EB3) in HeLa cells, as well as APXs triggered by the EGFP signals. EB3 binds to the plus end of the microtubule during tubulin polymerization.^[^ ^14^ ^]^ The EB3‐EGFP signals reveal highly dynamic tubulin polymerization processes in HeLa cells and highlight RPOC for tracking moving targets for optical treatment (Movies S1.1–S1.3, Supporting Information). The system exhibits a diffraction‐limited spatial resolution of ≈300 nm, with a similar spatial precision for optical manipulation (Figure S5 and Note S4, Supporting Information). Oversampling is used in this study, which gives the pixel size of 150 nm unless otherwise specified. Compared to the previous femtosecond RPOC,^[^ ^13^ ^]^ the CW‐RPOC system gives much higher sensitivity and lower photobleaching effect for the EGFP signals.

### 2.2 Organelle‐Specific Blue Light Interaction and Cell Response

In the first application, we explore using CW‐RPOC to precisely cast blue light only at desired organelles and monitor organelle‐specific cell responses. Studying how short‐wavelength light interacts with cells helps elucidate the mechanism of phototoxicity and cell stress under radiation. It is known that short‐wavelength blue light induces reactive oxygen species (ROS) in cells.^[^ ^15^, ^16^ ^]^ ROS are highly reactive molecules containing oxygen atoms with an unpaired electron. While ROS are natural byproducts of metabolic processes and are well‐regulated in live cells,^[^ ^17^, ^18^, ^19^ ^]^ increased ROS generation induced by pathological transitions or external stimuli can cause oxidative stress that significantly affects cell functions.^[^ ^20^, ^21^, ^22^ ^]^ Depending on the location of perturbation, the extent of blue light impact on cellular pathways is different. For instance, ER‐targeted photodynamic therapy is observed to be more efficient than using other organelle‐targeting photosensitizers.^[^^]^ Existing methods of treating cells with blue light lack subcellular spatiotemporal control, making it difficult to understand the impact of blue light on different organelles. With the CW‐RPOC, we can cast a 405 nm laser solely on organelles of interest with high spatial precision and well‐controlled dose, and quantify dosage‐dependent cell responses.

**Figure 2:**
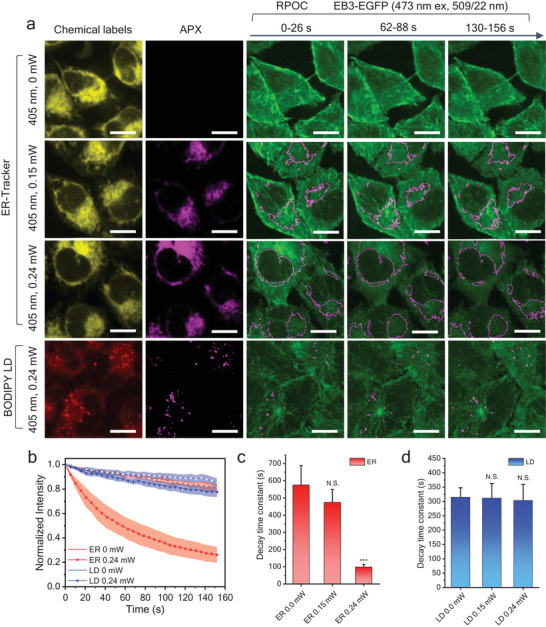
Organelle‐specific blue light interaction using CW‐RPOC. a) Representation of chemical selection channels, active pixels (APX) selected, and time‐dependent EB3‐EGFP signals from the readout channel. In the first three rows, the endoplasmic reticulum (ER) is labeled using ER‐Tracker. In the fourth row, lipid droplets (LDs) are labeled using BODIPY. To highlight ER‐selected APX areas, outlines of the APXs are overlaid with EGFP signals. Averaged EB3‐EGFP images from five frames acquired in 26 s are displayed for the three time points. b) Time‐dependent EB3‐EGFP signal change for cells in different treatment conditions. c,d) The decay time constants of EB3‐EGFP signals for cells when APXs are selected on c) ER and d) LDs. Scale bars are 10 µm. For statistical analysis, *** *p* < 0.001, N.S. *p* > 0.05.

HeLa cells with a stable EB3‐EGFP expression are used for this study. Fluorescent organelle markers are excited by either the 589 nm (10 µW) or 473 nm (36 µW) laser to locate organelles of interest as optical treatment targets. The 405 nm laser is used to directly perturb organelles of interest. The EGFP signals, excited by the 473 nm laser (36 µW), are used to visualize tubulin polymerization dynamics and quantify cell responses to localized blue light treatment. Using ER‐Tracker, APXs can be selected only at ER locations. The ER‐Tracker signals, APXs, and EB3‐EGFP fluorescence images during RPOC are shown in **Figure** **2**a. A significant EB3‐EGFP signal decrease is observed when a 240 µW 405 nm laser is shone on ER with a pixel dwell time of 20 µs (Figure 2a). Such a significant decrease is not observed in the control group (no APX) and treating ER with 150 µW 405 nm laser (Figure 2a). When selecting APX on LDs using BODIPY LD labeling and applying the same power of 405 nm laser uniquely at LDs, no considerable EB3‐EGFP signal decrease is detected (Figure 2a). Averaged time‐dependent EB3‐EGFP fluorescence signals from cells in each condition are normalized to compare trends in intensity change (Figure 2b). The curves are fit using exponential functions to obtain the decay time constants of each condition (Figure 2c,d). The results reveal that a substantial EB3‐EGFP signal decrease is associated with 240 µW 405 nm interacting with ER, not LDs. Aside from the EGFP signal decrease, the 240 µW 405 nm laser interaction with ER causes a faster disruption of cellular tubulin polymerization compared to other cases, which can be seen in Movie S2, Supporting Information. This EGFP signal decrease is not induced by photobleaching. The 405 nm laser is not in the excitation band of EGFP and thus has a minimum photobleaching effect on the EGFP. To confirm this, we used RPOC to cast 240 µW 405 nm laser only on the dynamic EB3 molecules (Figure S6, Supporting Information). Despite perfectly overlapping with the EGFP signals, the 405 nm laser does not induce a significant EGFP signal decrease. The relatively long pixel dwell time for the 473 nm laser used in RPOC (20 µs) induces a very slow EB3‐EGFP signal decrease (Figure 2b, and Figure S6b, Supporting Information). Such a signal change is not detected when the excitation laser dosage is reduced (Figure S7, Supporting Information).

**Figure 3:**
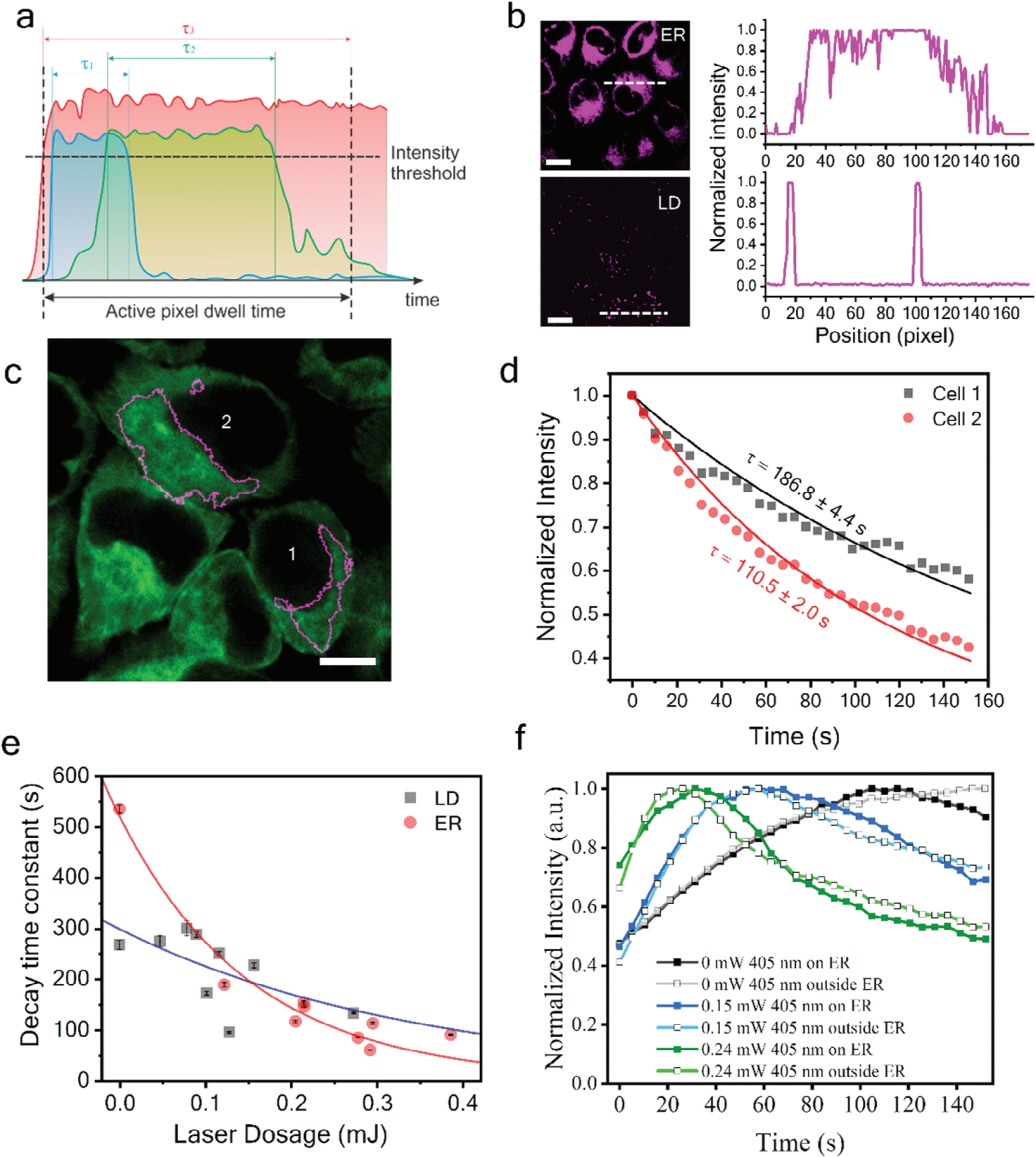
Laser‐dosage‐dependent cell responses to organelle‐specific generation of reactive oxygen specie. a) An illustration of different optical signals in an APX and the corresponding time (τ_1_, τ_2_, τ_3_) when signal levels are above the intensity threshold. b) Example images of APX on ER and LD and the corresponding APX line profiles. c) EB3‐EGFP signals of HeLa cells and the outlines of ER‐APXs selected for two cells. d) Time‐dependent EB3‐EGFP signal changes for cells 1 and 2 in panel (c). Dots are experimental results; lines are fitting curves using an exponential decay function. e) EB3‐EGFP signal decay time constant plotted against the 405 nm laser dosage for LD and ER. Dots are experimental results; lines are fitting curves using an exponential function. f) Normalized H2DCFDA signals on and outside of ER for HeLa cells treated with different power of 405 nm laser on ER. Scale bars are 10 µm.

Figure 2a implies that cell responses to localized blue light treatment are laser‐dosage‐dependent. To correlate EGFP signal changes to laser dosage, a quantitative analysis method is developed. The laser dosage can be quantified using APX intensity. As shown in **Figure** **3**a, APX intensity measures the 405 nm laser power multiplying the total time of the fluorescence signals exceeding the intensity threshold in the APX. If the target fluorescence signal is strong, the signal would exceed the threshold for a longer time, resulting in higher APX intensity. The maximum APX intensity is measured when the fluorescence signal is above the threshold throughout the entire pixel dwell time. Figure 3b shows example APX images and the corresponding APX intensity profiles at dash lines when ER and LDs are selected. Integration of APX intensity in a cell quantifies the dosage of 405 nm laser received by the cell. The quantification procedures are detailed in the Experimental Section. The ER boundaries of two HeLa cells are shown in Figure 3c. The laser dosage of cell 1 is around 123 µJ, which is much lower than 296 µJ for cell 2. Despite both being treated on ER, cell 1 and cell 2 show different intensity decay profiles due to the laser dosage difference (Figure 3d). A higher laser dosage tends to induce a faster decay of EB3‐EGFP signals (Figure 3d). We quantify 405 nm laser dosage for selective treatment of ER and LD in each cell and plot their correlations with the corresponding EGFP signal decay (Figure 3e). The difference in such correlations for ER and LD indicates that the 405 nm laser has different impacts on different organelles. In general, the total area of the ER is much larger than the total area of LDs in cells. However, the APXs on ER can be tuned by the intensity threshold so that in some cells, especially when the LDs are rich, the total laser dose on LDs can be comparable or even exceed that on ER. Note that for the untreated case (0 laser dosages), LD‐labeled cells have a faster EGFP signal decay compared to ER‐labeled cells. This is likely due to the higher cytotoxicity and functional perturbation induced by BODIPY compared to ER‐Tracker. However, as the 405 nm laser dosage increases, the same laser dosage on ER causes a faster EGFP signal decrease than on LDs.

Aside from the decrease of EB3‐EGFP signals, our study by treating cells using a 405 nm laser confirms blue‐light‐induced disruption of tubulin polymerization in live cells (Figure S7 and Note S4, Supporting Information). The decrease of EGFP signals and tubulin polymerization when ER is treated with a sufficient dose of blue light is likely due to ROS generation. It is well‐documented that blue light can induce ROS that impacts cellular functions^[^ ^15^, ^16^ ^]^ such as decreasing microtubule growth.^[^ ^24^ ^]^ To monitor ROS generation during RPOC, we treated HeLa cells (no EGFP transfection) with 2′,7′‐dichlorodihydrofluorescein diacetate (H2DCFDA), a fluorescent free‐radical sensor. ROS generated in cells increases the fluorescent signals of H2DCFDA. We found that without 405 nm radiation, ROS can be generated at a slow rate (Figure 3f, and Figure S8a,b, Supporting Information), especially in the mitochondria. Such an increase in ROS is likely due to the photosensitization effect of ER‐Tracker induced by the 589 nm laser. However, when the 405 nm laser is cast on the ER, the ROS generation speed is significantly increased (Figure 3f, and Figure S8c–f, Supporting Information) both on and outside the ER APX area. A higher 405 nm laser power on ER gives a faster rise in the ROS. The subsequent H2DCFDA signal decrease is likely due to the damage of the fluorophore by the excess amount of ROS. The escalated EB3‐EGFP signal loss in Figures 2 and 3 when ER is treated with blue light is likely mediated by a mechanism related to ER. It has been reported that an excess amount of ROS in ER can cause the leaking of calcium into the cytosol. Such released calcium can induce mitochondrial dysfunction and a cascade of apoptotic events.^[^ ^8^, ^25^ ^]^ Our results indicate that cell response to localized blue light treatment is organelle‐dependent. Compared to LDs, ER is a more responsible organelle for the blue light‐induced loss of EGFP signal and disruption of tubulin dynamics.

**Figure 4:**
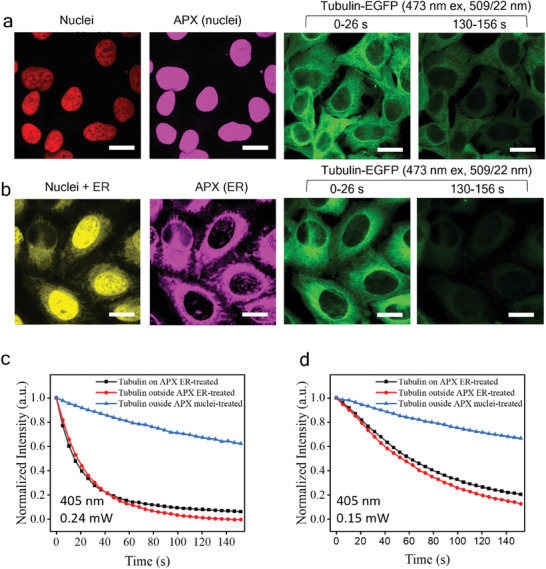
Comparing 405 nm laser interaction with nuclei and ER using CW‐RPOC. a) Representation of nuclei mCherry signals, APXs selected, and time‐dependent tubulin‐EGFP signals from the readout channel. Averaged tubulin‐EGFP images from five frames acquired in 26 s are displayed for the two time points. b) Similar to panel (a) but selecting APX on ER. ER are labeled using the ER‐Tracker Red. c) Examples of tubulin‐EGFP signal changes as a function of time for areas on or outside APX when nuclei or ER are treated with 240 µW 405 nm blue laser. d) Similar to panel (c) but using 150 µW 405 nm laser for organelle interactions. Scale bars are 20 µm.

Besides ER and LD, we also studied the impact of blue light on cell nuclei. Available fluorescent nucleic acid stains are not suitable for our study because of their high phototoxicity. Instead, EGFP‐alpha‐tubulin/H2B‐mCherry Hela cells are used for this study. The nucleus signals are excited using the 589 nm lasers for APX selection on nuclei. The 405 nm laser is applied to directly perturb the nuclei. The tubulin‐EGFP signals, excited by the 473 nm laser, are used to measure the cell responses. To compare nuclei treatment with ER treatment, the ER can be labeled using ER‐Tracker Red which shows up in the same channel as the nuclei but with lower signal levels. Utilizing the advanced comparator circuit, we can select an intensity range for RPOC and cast a 405 nm laser only on ER to compare with the nuclei treatment. For the control group (0 µW 405 nm laser), tubulin‐EGFP signals show a similar intensity change profile to the EB3‐EGFP signals (Figure S9, Supporting Information). We also compared the EB3‐EGFP and tubulin‐EGFP signal loss during 1.1 mW 405 nm laser treatment when the laser is on for all pixels (Figure S10, Supporting Information). These results suggest that EGFPs in the two cell lines used in this study have similar responses to blue light treatment. Then, we compare the selective treatment of nuclei (**Figure** **4**a) and ER (Figure 4b) using the 405 nm laser. Despite the laser doses being similar, the ER‐treated cells show a much faster EGFP signal loss at both 240 µW and 150 µW treatment laser power, compared to nuclei‐treated cells (Figure 4c,d). We also compared tubulin‐EGFP signals on and outside APXs (Figure 4c,d and Figure S11, Supporting Information), which show a similar change when ER is treated. This further confirms the escalated EGFP signal decrease during ER treatment is not induced by photobleaching.

Collectively, these studies demonstrate that RPOC allows laser interaction with selected cellular targets with high precision and selectively, and simultaneously monitoring cellular responses to localized perturbation with high sensitivity.

### 2.3 Precise Inhibition of Biomolecular Activities at Subcellular Targets

**Figure 5:**
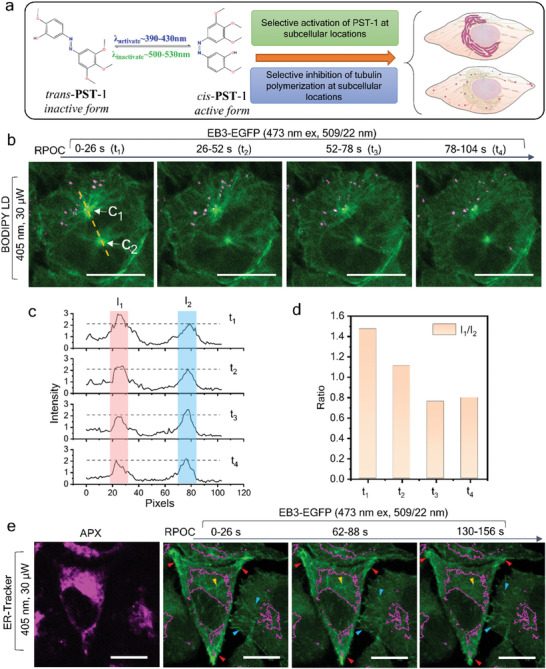
Precision inhibition of tubulin polymerization at subcellular compartments. a) Interconversion of *trans*‐PST‐1 and *cis*‐PST‐1 upon stimulation by blue and green laser wavelengths. An illustration of selective activation of PST‐1 only on ER and around LDs by RPOC. b) EB3‐EGFP signals over time with APX displayed in magenta. Cells are treated with inactive PST‐1 which is only activated at APXs. APXs are associated with centrosome C_1_ but not C_2_. Averaged EB3‐EGFP images from five frames acquired in 26 s are displayed for the four time points. c) The change in the intensity of EB3‐EGFP signals along the dashed line in panel (b) for two centrosomes. d) The ratio of *I_1_* over *I_2_* as a function of RPOC activation time. e) APX selected by ER‐Tracker and EB3‐EGFP signals at different time points when PST‐1 is selectively activated on ER. The ER APX areas are outlined in EB3‐EGFP images. Scale bars are 20 µm.

Apart from directly perturbing cellular changes by lasers, RPOC can selectively control the activities of photoswitchable compounds to inhibit chemical processes exclusively at subcellular targets. A photoswitchable tubulin polymerization inhibitor photostatin (PST‐1) was synthesized as previously reported.^[^ ^13^, ^26^ ^]^ The photoswitchable active *cis‐* and inactive *trans‐*isomers of PST‐1 are shown in **Figure** **5**a. Selective photoactivation of PST‐1 allows for inhibition of tubulin polymerization at selected targets in cells (Figure 5a). To rule out the potential impact of blue‐light‐induced ROS and EGFP signal loss, 30 µW of 405 nm laser is used. Such a low laser dosage does not induce detectable decreases in EB3‐EGFP signals. Furthermore, the inhibition of tubulin polymerization does not decrease EB3‐EGFP signals integrated over a whole cell (Figure S7 and Notes S4 and S9, Supporting Information). This is because the depolymerized EB3 molecules are released from the microtubule into the cytosol and remain fluorescent.

Due to the highly dynamic signature of EB3‐EGFP signals, it is hard to quantify the inhibition of tubulin polymerization at single LDs. Instead, we select centrosomes, which are EB3 abundant and are constantly bright in fluorescent images, to quantify the inhibition process. Figure 5b represents a field of view of HeLa cells with two centrosomes detected. Cells are incubated with inactive PST‐1 before RPOC. LDs are labeled using BODIPY and PST‐1 molecules are activated at the LD areas, which are close to and present on one of the centrosomes (C_1_). The other centrosome (C_2_), on the other hand, is distant from LD APXs. Time‐lapse EB3‐EGFP intensity shows a decrease in C_1_ together with no change for C_2_ during RPOC (Figure 5b,c, and Movie S3, Supporting Information). The ratio of centrosome intensities I_1_/I_2_ over time further highlights such a change (Figure 5d), which is due to selective inhibition of tubulin polymerization at C_1_ but not C_2_. These results demonstrate that even inside a single cell, RPOC can inhibit biomolecular activities at subcellular compartments and visualize cell responses simultaneously.

**Figure 6:**
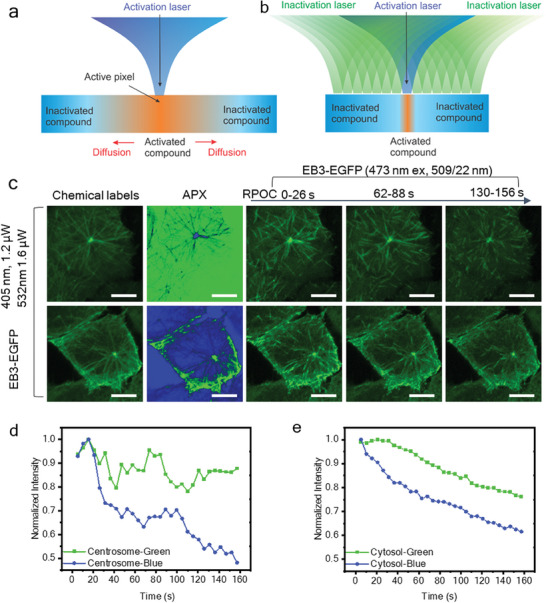
Subcellular control of photoswitchable inhibitor activities. a) A schematic representation of the diffusion of activated photoswitchable compounds to other subcellular locations in the absence of an inactivation laser. b) A schematic representation of the inactivation of an activated compound in all locations other than desired target sites using the inactivation laser. c) Chemical labels (EB3), APXs, and EB3‐EGFP signals during selective activation/inactivation of PST‐1 at different subcellular locations. Top row: Inactivation of PST‐1 only at the centrosome while activating it elsewhere. Bottom row: activation of PST‐1 only at the centrosome and the cell boundary while inactivating it elsewhere. Green in the APX channel indicates APXs for 532 nm, while blue in the APX channel indicates APXs for 405 nm. Averaged EB3‐EGFP images from five frames acquired in 26 s are displayed for the three time points. d) EB3‐EGFP signals at centrosomes when the centrosome is treated with blue or green light. Cells are incubated with inactivated PST‐1 before RPOC. e) Similar to panel (d) but for cytosol areas excluding centrosomes and other EB3‐dense areas. Scale bars are 10 µm.

Using ER‐Tracker to select APX on ER, RPOC can activate PST‐1 and inhibit tubulin polymerization only in the ER area, as shown in Figure 5e. Compared with the cell protrusion area (red arrows), EB3 comets on ER (yellow arrows) decrease more notably (Movie S4, Supporting Information). Note that when only using blue light for PST‐1 activation, the activated compound can gradually diffuse into areas outside APXs, and eventually lead to inhibition of tubulin dynamics of the whole cell, as illustrated in **Figure** **6**a. To prevent such active drug diffusion, the 532 nm laser can be utilized to inactivate PST‐1 outside APXs of the 405 nm laser (Figure 6b). This function can be achieved by the CW‐RPOC system since the 405 and 532 nm lasers are separately controlled by two AOMs and different channels of the comparator circuitry. Here, 1.2 µW 405 nm and 1.6 µW 532 nm lasers are used for optical manipulation. Figure 6c shows EB3 signals, APXs from two lasers, and time‐lapse EB3 signal changes over time. In the top panels, PST‐1 is mostly activated in the EB3‐dense area (mostly centrosome) and inactivated elsewhere, while the bottom panels reverse the activation/inactivation locations. Comparing EB3‐EGFP signals from centrosomes, we found that PST‐1 activation on centrosomes by blue light shows a more pronounced signal decrease compared to that is inactivated by green light (Figure 6d, and Movies S5 and S6, Supporting Information). Such a change is also observed in the cytosol areas with low EB3‐EGFP signals (Figure 6e). The decrease of EGFP signals during 532 nm laser treatment in cells is induced by the photobleaching of EGFP by the 473 nm laser. The inactivation of PST‐1 by 532 nm laser increases the concentration of EB3 in the less polymerized locations. This process compensates for the photobleaching of EGFP, resulting in a flat plateau in the first 40 s in Figure 6e. For EB3‐dense centrosomes, such compensation is more efficient due to the lower dosage and interaction area of the 532 nm laser, giving a much slower signal decay as shown in Figure 6d. Due to the selection of a much smaller area, the centrosome EGFP signal changes are more susceptible to the impact of tubulin dynamics, resulting in higher fluctuations in the intensity change (Figure 6d). This study exemplifies that CW‐RPOC can selectively activate photoswitchable inhibitors and enable subcellular site‐specific inhibition of biomolecular processes at high spatiotemporal precision.

## 3 Discussion

Conventionally, it is impossible to precisely control chemical reactions and enzyme activities in cells at submicron precision. CW‐RPOC provides a new approach using lasers to manipulate biomolecular processes at high spatial precision and in real time. We demonstrate the selective interaction of blue light at different doses with selected organelles and quantify the differences in cell responses. Furthermore, subcellular‐localized inhibition of tubulin polymerization at selected organelles is achieved. RPOC does not require a priori knowledge of chemical targets and accomplishes target detection, decision‐making, and optical treatment simultaneously. This is especially advantageous for tracking and treating entities with highly dynamic signatures such as LDs, mitochondria, lysosomes, tubulin, actin, and other proteins with high mobility.

Compared to the femtosecond RPOC prototype,^[^ ^13^ ^]^ CW‐RPOC greatly reduces the cost and complexity of the system and makes RPOC a technology fully compatible with confocal fluorescence microscopes for broad applications in biological sciences. A detailed cost comparison can be found in Note S2, Supporting Information. Furthermore, single‐photon excitation by CW lasers gives much stronger fluorescence signals and less photobleaching than the two‐photon excitation fluorescence using femtosecond lasers. This opens RPOC to most existing fluorescent proteins and dyes to identify and track organelles, proteins, and metabolites for both sensitive chemical detection and readout. Moreover, we demonstrate simultaneous control of two laser wavelengths with high spatiotemporal precision for optical manipulation. This approach not only adds enhanced flexibility for optical manipulation but also reduces activated photosensitive inhibitors from diffusing to unwanted locations.

In Figures 2, 3, 4, the EGFP signals from the entire cell are analyzed and compared. Only the ROS generation by the 405 nm laser and the photobleaching by the 473 nm laser contribute to the EGFP signal decrease. The latter is measured in each treatment condition as the control group for comparison. The ROS generated by the 405 nm laser can accelerate the EGFP signal decay. The 405 and 589 nm lasers do not cause a detectable photobleaching effect, as discussed in Note S9 and Figure S6, Supporting Information. In Figures 5 and 6, the EGFP signals from subcellular locations are analyzed and compared. Although the inhibition of microtubule polymerization does not decrease the overall EGFP signals of the entire cell (Figure S7, Supporting Information), it affects the subcellular EGFP signals due to the redistribution of the EB3 and tubulin molecules. The power of 405 and 532 nm lasers is kept very low in this case to avoid photobleaching or ROS generation. A more detailed discussion can be found in the Supporting information (Note S9, Supporting Information).

In this work, only 405 and 532 nm lasers are used for optical manipulation. Applying more lasers and AOMs would allow more fluorescent labels for organelle detection and additional laser wavelengths for opto‐control. Aside from EGFP, other fluorescent proteins transfected to cells or fluorescent labels can be applied as the readout for RPOC. In addition, cell behaviors such as proliferation, migration, and differentiation can also be used as readouts to study long‐term cell responses.

We demonstrated precision control of tubulin dynamics in live cells using PST‐1. Photoswitchable inhibitors of other cytoskeletal proteins or cellular enzymes can be paired with CW‐RPOC for precision manipulation of a wide range of biomolecules in cells.^[^ ^27^, ^28^, ^29^, ^30^, ^31^, ^32^, ^33^ ^]^ This approach would lead to new insights into cellular function through location‐specific manipulation of biomolecular activities, subcellular activation of drugs, and precisely controlled release.

By shedding light on previously inaccessible aspects of cellular processes, CW‐RPOC opens up new avenues for understanding drug‐target interactions, exploring site‐specific biomolecular activities, and facilitating precise control over chemical release. On a larger scale, RPOC might bring about photodynamic therapy and treatment strategies with higher spatial and chemical precision and lower side effects.

## 4 Experimental Section

### The CW‐RPOC Technology

A detailed schematic of the CW‐RPOC system is shown in Figure 1. The system incorporated four diode‐pumped solid‐state lasers (CNI Laser), which were collinearly combined using three dichroic beamsplitters (DMLP567, DMLP505, and DMLP425, Thorlabs). Each laser was collimated by a pair of lenses, and the laser power was adjusted using variable neutral density filters (54–081, Edmund Optics). Laser 1 (*λ* = 405 nm, 100 mW) and laser 3 (*λ* = 532 nm, 100 mW) were action lasers dedicated to optical manipulation and were independently controlled by two AOMs (M1205‐P80L‐0.5 with 532B‐2 driver, Isomet). A small portion of both lasers 1 and 3 were directed to separate photodiode detectors (PDA10A2, Thorlabs) for visualization of APXs. Laser 2 (*λ* = 473 nm, 100 mW) was used for exciting EGFPs in living cells. Laser 4 (*λ* = 589 nm, 100 mW) was employed for exciting chemical targets for opto‐control. The laser beams were expanded separately by different lens pairs to ≈2.5 mm in diameter. For lasers 1 and 3, the lenses were placed after AOMs. The combined beams passed through a polarizing beamsplitter (PBS251, Thorlabs) and a quarter‐wave plate before entering a 2D galvo scanner set (Saturn‐5 system, ScannerMAX). The laser beams were further expanded by a pair of lenses to fill the entrance of a water‐immersion apochromatic objective lens (UPlanSApo‐S, 60×, NA = 1.20, Olympus) mounted on an inverted microscope (IX73, Olympus). Sample positioning was achieved using a 3D translational stage (H117 with Motor Focus Drive and ProScan III system, Prior).

The polarizing beam splitter directed the back‐reflected fluorescence signals to two PMTs (H7422‐40, Hamamatsu). Confocal detections were enabled by placing two pinholes (P300HK, Thorlabs) at the sample conjugate focal plane. The fluorescence signals from EGFP were detected by the PMT in the readout channel, which was equipped with a bandpass filter centered at 509 nm (FF01‐509/22, Semrock). The fluorescence signals from mCherry proteins or organelle labels were detected by the PMT in the organelle selection channel, which utilized a bandpass filter centered at 642 nm (ET642/80m, Chroma Technology Corporation). These two channels were separated by a long‐pass dichroic beam splitter with a cutoff wavelength at 552 nm (FF552‐Di02, Semrock). The absorption and emission spectra of BODIPY and ER‐Tracker used in this work are shown in Figure S12a,b, Supporting Information. The laser wavelengths and filter passband windows for signal detection are illustrated in Figure S12c, Supporting Information. The PMT output currents from both channels were converted to voltage and amplified using two preamplifiers (PMT4V3, Advanced Research Instruments Corporation) before being sent to the data acquisition system (PCIe‐6363 paired with BNC‐2110, National Instruments) and two‐channel comparator circuit. A custom multichannel image acquisition software based on LabVIEW was employed for image display, acquisition, and APX detection. A two‐channel comparator circuit box was designed to achieve APX selection and closed‐loop feedback control. The detailed design and functions of the comparator circuitry can be found in Note S1, Supporting Information. The TTL serial commands controlled the two AOMs separately. This box allowed for the selection of APX from two different lasers (405 and 532 nm) based on the fluorescence channels either separately or in a logical computation manner.

### Laser Dosage Measurement

The APX signals from the 405 nm laser, which was directly captured by a photodiode, provide real‐time feedback on the laser dosage. If the fluorescence signal was above the threshold for the entire pixel dwell time, the maximum APX intensity *I_max_* was measured, which could be achieved at the APX intensity saturation during RPOC or constantly turning on the AOM using the comparator circuit box. *I_max_* was used to normalize the laser dosage on the sample.

For the time‐lapse analyses performed in this study, multiple frames were acquired during RPOC. To quantify the laser dosage for the entire interaction time for a cell, the EB3‐EGFP signals in the readout channel were used to manually outline individual cells. The cell mask was then projected to the averaged APX image (over acquired *N* frames) for quantification. The average intensity *I_ave_* and mask area *A* were measured for each cell. An area outside of the cell in the average APX image was selected to measure the background intensity *I_bg_*. The opto‐control laser at the sample had an average power of *P*. The pixel dwell time for RPOC and imaging was *T*. The laser dosage for each cell, which has a unit of J, can be calculated using equation (1). All image processing, mask selection, and quantifications were performed using ImageJ built‐in functions.

(1) $$D=\frac{A\times \left(I_{ave}-I_{bg}\right)\times P\times T\times N}{I_{max}-I_{bg}}$$

### Quantitative Image Analysis

Pseudo‐color‐labeled images of molecular or organelle targets, APXs, and EB3‐EGFP were processed using ImageJ. In Figures 2 and 4, 5, 6, EB3‐EGFP or tubulin‐EGFP images were averaged in different time windows for display. Chemical labels and APX images were averaged from 0–156 s for display. To obtain time‐dependent EB3‐EGFP signal changes, single cells were manually outlined, and fluorescence signals gated in each cell were integrated for each frame. The integrated fluorescence intensity for each cell was plotted as a function of time to quantify cell responses. The fluorescence intensity decay curves were subtracted by background intensities and normalized by dividing the maximum value. The normalized intensity decay curves could be fit using an exponential decay function.

(2) $$I=I_{0}+Ae^{-t/\tau }$$

The decay curve with the fastest time constant was used to obtain *I_0_* and *A*, which were maintained constant for the fitting of other conditions. The decay time constant τ was compared for different conditions in Figures 2 and 3. For the Student's *t*‐test in Figure 2, five cells were quantified in each condition. APX intensity profiles in Figures 3, 4, 5 are plotted using an ImageJ built‐in function.

### Cell Preparation

HeLa Kyoto EB3‐EGFP cells and HeLa Kyoto EGFP‐alpha‐tubulin/H2B‐mCherry cells were purchased from Biohippo Inc. HeLa cells (no fluorescent protein transfection) were purchased from ATCC. Cells were cultured in Dulbecco's modified Eagle medium (30‐2002, ATCC) with 10% fetal bovine serum (30‐2021, ATCC) and 1% penicillin/streptomycin (15140122, Thermofisher Scientific). The cells were seeded in 35 mm glass‐bottom dishes (MatTek Life Sciences) with 2 mL culture medium and then incubated in a CO_2_ incubator at 37 °C and 5% CO_2_ concentration. Cells were grown to about 50% confluency and directly used for live‐cell imaging.

### PST‐1 and Cell Treatment with PST‐1

The synthesis of PST‐1 could be found in a previous publication.^[^ ^13^ ^]^ PST‐1 was dissolved in dimethylsulfoxide at 2 mM concentration to prepare the stock solution. Before treating the cells, the PST‐1 stock solution was illuminated with a 532 nm laser (CNI laser) for 5 s to convert PST‐1 into the *trans*‐ inactivated form. Cells were treated with inactivated PST‐1 at a final concentration of 4 µM for 15  min before RPOC. The 405 nm laser (CNI laser) in the CW‐RPOC system was used to selectively activate PST‐1 solely at desired locations by CW‐RPOC.

### Fluorescent Labeling of ER and LDs

HeLa Kyoto EB3‐EGFP cells were first seeded in glass‐bottom dishes and cultured overnight to reach a confluency of around 50–70%. ER‐Tracker Red (E34250, Thermo Fisher Scientific) was added to the culture medium with a final concentration of 1 µM. Similarly, LDs were labeled by BODIPY 505/515 nm (25893, Cayman Chemical) and added to the culture medium with a final concentration of 5 µM. The cells were then incubated for 30 min at 37 °C and 5% CO_2_ concentration before RPOC. For BODIPY labeling, cells were washed twice with a warm culture medium after BODIPY incubation before RPOC. The absorption spectra of the dyes were measured using a UV–Vis spectrometer (GENESYS 50, Thermo Fisher Scientific). The emission spectra of the dyes were measured using a fluorospectrometer (NanoDrop 3300, Thermo Scientific)

### ROS Measurements using H2DCFDA

HeLa cells were first seeded in glass‐bottom dishes and cultured overnight to reach a confluency of around 50–70%. H2DCFDA (D399, Thermo Fisher Scientific) and ER Tracker Red were added to the culture medium with a final concentration of 10 and 1 µM respectively. The cells were then incubated for 30 min at 37 °C and 5% CO_2_ concentration before RPOC or imaging.

### Statistical Analysis

The biological studies were repeated multiple times for all experiments. For Figure 2, five cells were analyzed in each condition to obtain statistical results. The cells were selected from or close to the center of the images. Partially exposed cells and initially rounded‐up cells were not taken into consideration during the analysis. A 2‐tailed 2‐type *t*‐test was performed and the *p*‐value for each case was determined. Any *p*‐value of less than 0.001 was considered highly significant (***) and any *p*‐value greater than 0.05 was regarded as non‐significant (N.S.).

## Conflict of Interest

The authors declare no conflict of interest.

## Author Contributions

B.D. and S.H.M. contributed equally to this work. B.D. and M.G.C. built the confocal microscope and RPOC platform. K.B. built the galvo scanning system. B.D. and S.H.M. performed measurements on biological samples and quantitative image analysis. S.H.M. and S.M. cultured cells. S.H.M. performed chemical treatment of cells. M.S.C. designed and built the comparator circuit. G.C. was involved in the design of the comparator circuit. K.J.M. measured the RPOC system response time. C.Z. designed and supervised the project. C.Z., B.D., and S.H.M. wrote the manuscript.

## Supporting information

Supporting Information

Supplemental Movies 1

Supplemental Movies 2

Supplemental Movies 3

Supplemental Movies 4

Supplemental Movies 5

Supplemental Movies 6

Supplemental Movies 7

Supplemental Movies 8

## Data Availability

The data that support the findings of this study are available from the corresponding author upon reasonable request.
