# Supplementary material for: Spatiotemporally Precise Optical Manipulation of Intracellular Molecular Activities
Source: Adv Sci (Weinh). 2024 Jan 26;11(13):2307342. doi: 10.1002/advs.202307342 (PMC10987104; doi:10.1002/advs.202307342)
Supplement: Supplementary file 1 — Supporting Information [file ADVS-11-2307342-s002.pdf]

ADVANCED SCIENCE
Open Access

Supporting Information

for *Adv. Sci.*, DOI 10.1002/advs.202307342

Spatiotemporally Precise Optical Manipulation of Intracellular Molecular Activities

*Bin Dong, Shivam Mahapatra, Matthew G. Clark, Mark S. Carlsen, Karsten J. Mohn, Seohee Ma, Kent A. Brasseale III, Grace Crim and Chi Zhang**

Supporting information

Spatiotemporally precise optical manipulation of intracellular molecular activities

Bin Dong[#1], Shivam Mahapatra[#1], Matthew G. Clark[1], Mark S. Carlsen[1], Karsten J. Mohn[1], Seohee Ma[1], Kent A. Brasseale III[1], Grace Crim[1], and Chi Zhang[1,2,3]*

[1]Department of Chemistry, Purdue University, 560 Oval Dr., West Lafayette, IN 47907, USA

[2]Purdue Center for Cancer Research, 201 S. University St., West Lafayette, IN 47907, USA.

[3]Purdue Institute of Inflammation, Immunology, and Infectious Disease, 207 S. Martin Jischke Dr., West Lafayette, IN 47907, USA.

Supplementary Notes

1. Two-channel comparator circuitry

The workflow of the two-channel comparator circuit is shown in **Figure 1b**. A photograph of the circuit box is shown in **Figure S1**. Two signal inputs can be connected to two separate detection channels and compared with separate preset thresholds. The threshold can be selected from a manual selection knob or input from separate BNC ports. Switches are used to select manual thresholds or analog/digital input thresholds for signal comparison. If the intensity of the fluorescent signal exceeds the respective threshold, a '1' Transistor-Transistor Logic (TTL) command is sent to the output. Otherwise, a TTL '0' is the output command. Invert functions can be selected to invert the output commands. This output can be directly sent to AOMs via comparator outputs to direct or stop the 1st order laser beam to the sample. In this paper, thresholds are selected manually. Copies of the input thresholds are available as comparator circuit outputs.

In addition, logic gates are available to make logic computations before sending the TTL to the AOM via logic outputs. AND, OR, and NOT functions are available separately for both of the two logic outputs. This function allows us to make higher-order decisions based on signals from two detectors. AOMs can also be controlled to be constantly on or constantly off by using AOM control switches before the logic outputs. Aside from output ports for AOM control, buffered output replicates the input signals which can be connected directly to the image acquisition channels to display images. Using a Tee connector at the TTL outputs, APXs can be directly visualized while driving AOM with TTL signals.

Figure S2a, b shows two connection methods to achieve the same APX for both blue (405 nm) and green (532 nm) lasers for opto-control. **Figure S2c** shows a fluorescence image of pollens excited using the 589 nm laser, and the corresponding APXs of the green and blue lasers selected using the connection in panel b. Note that both green and blue lasers can be simultaneously turned on only at the pollens. Here, only the input channel 1 is used. **Figure S3a, b** shows the connection and selection of green APXs on pollens while blue APX outside pollens simultaneously. By flipping two switches at the inverters, the APXs from the green and blue lasers can be swapped (**Figures S3c, d**). The thresholds for

APXs from the two lasers are separately tunable if the input signal is split by a Tee connector and connected to both inputs.

2. The system cost of CW-RPOC

The use of CW lasers significantly reduces the cost of RPOC technology compared to its femtosecond-laser-based predecessor. The cost for the 405 nm, 473 nm, 532 nm, and 589 nm lasers, all with 100 mW output power, is ~$13,000 in total. Furthermore, since only < 1 mW laser power is typically used for RPOC and optical imaging, lasers with 30-50 mW are powerful enough for the CW-RPOC. Therefore, the total laser cost can be further reduced to < $10,000. Compared to the ~$200,000-$250,000 InSight X3+ used in the stimulated Raman scattering (SRS) system, the laser cost of the CW-RPOC is only 4-5% of the femtosecond-RPOC.

The cost of the microscope frame, high NA objective lens, 3D motorized sample stage, and optical components are ~$60,000 in total for CW-RPOC and ~$80,000 for the femtosecond RPOC, not including the stage-top hypoxia incubator. The major cost difference is due to the use of lock-in amplifiers, intensity modulation, spectral chipping, and optical delay scanning of the SRS modality. Therefore, considering the total system cost, the CW-RPOC is only ~21-25% of the femtosecond-RPOC system. Here, the price includes a commercial microscope frame and a high-performance motorized 3D sample stage. If a simple microscope frame and manual 2D sample stage are used, the cost of the total CW-RPOC system can be reduced to ~$30,000 and the femtosecond-RPOC to ~$250,000-$300,000. The cost of the CW-RPOC is then only ~10-12% of the femtosecond-RPOC system.

3. The response time of CW-RPOC

The response time of the feedback system is measured using an oscilloscope. A signal pulse is sent from a function generator to both the oscilloscope and the comparator circuit. The latter generates a TTL '1' signal that controls the AOM. The 1st order laser from the AOM is acquired by a fast photodiode and sent to the oscilloscope. Comparing the time difference between the input signal and the response, the response time of the 405 nm laser is measured to be 580 ns (**Figure S4a**). Similarly, the response time of the 532 nm laser is measured to be 658 ns (**Figure S4b**). The delay is mostly generated by the AOM driver and AOM crystal response time. Such a speed, however, is much faster than the 10-20 μs pixel dwell time. The fast response time ensures simultaneous chemical detection and optical manipulation at the same pixel.

4. The spatial resolution and opto-control precision of CW-RPOC

The spatial resolution is characterized using a fluorescence image from HeLa Kyoto EB3-EGFP cells (**Figure S5a**). Fluorescence signals are excited using the 473 nm laser at 30 μW. A signal intensity line profile is plotted along a line that crosses one of the smallest structures in the image (**Figure S5b**). A Gaussian fitting gives a full width at half maximum (FWHM) of ~309 nm. We, therefore, conclude that the spatial resolution of the CW-RPOC system using the 473 nm laser is 309 nm. All laser beams are collinearly combined and overfill the back aperture of the objective lens. Therefore, the spatial precision of RPOC using 532 and 405 nm wavelength lasers can be estimated to be 348 nm and 265 nm, respectively. The axial resolution of the system is estimated to be on the level of 500 nm ~ 1 μm. The resolution can be further improved using a higher NA objective lens. Due to a higher laser intensity at the focus, chemical changes induced by the lasers such as ROS generation or photoactivation, are more

efficient at the laser focus. Note that reducing the pinhole size can increase the axial resolution of the images but not the axial precision for the optical manipulation.

5. Impact of the 405 nm laser on tubulin dynamics studied by confocal fluorescence microscopy

To further explore the impact of blue lasers on the cellular EB3-EGFP signals and the tubulin polymerization process, we used a commercial confocal fluorescence microscope (LSM510, Zeiss) for imaging and simultaneous laser treatment. Note that the commercial confocal microscope can perform simultaneous imaging and laser treatment but cannot selectively direct lasers solely at selected organelles as RPOC. Therefore, lasers are interacting with all pixels in the field of view (FOV). Cells are treated with a 160 μW 405 nm laser for approximately 10 minutes. The laser used for the EB3-EGFP signal is 488 nm with 80 μW power on the sample. The pixel dwell time is 1.6 μs. No significant fluorescence signal loss is detected (**Figure S7a-c**) after treatment, but the tubulin polymerization process is stopped by the blue light. The 405 nm laser does not induce detectable photobleaching of EB3-EGFP but impacts the microtubule polymerization dynamics by eliminating all EB3 comets.

6. Measurement of reactive oxygen species

To measure the cellular reactive oxygen species (ROS) production, 2',7'-dichlorodihydrofluorescein diacetate (H2DCFDA) is used at a final concentration of 10 μM. HeLa cells are labeled with ER-Tracker Red and are constantly illuminated with 25 μW 473 nm laser and 10 μW 589 nm laser. We found that even without the introduction of a 405 nm laser, the H2DCFDA signals increase (**Figure S8a,b**). Such an increase in the H2DCFDA signals indicates the rise of ROS in cells. This process is likely due to the photosensitization effect induced by the 473 and 589 nm imaging lasers. The results also indicate a stronger ROS generated in mitochondria (**Figure S8b**). However, the H2DCFDA signal increase is much faster when ER is illuminated with the 405 nm laser (**Figure 3f**). This change suggests that the 405 nm laser is elevating the ROS significantly upon ER interaction. The decrease of the H2DCFDA signals after reaching the maximum is likely due to the photoinactivation of the fluorophore by the excess amount of ROS. Especially, the cells in the center of the image show a more significant H2DCFDA signal loss while the cells are the edges give a much slower signal decrease. The time-dependent intensity profiles of H2DCFDA on and outside ER APXs are very similar, indicating the rapid diffusion of ROS in cells.

7. Comparing EGFP response of different cell lines and at different subcellular locations

We found that cell labeling using ER-Tracker Red does not alter the tubulin-EGFP signal intensity changes in the control condition (i.e. only illuminating cells with 25 μW 473 nm laser and 10 μW 589 nm laser), as shown in **Figure S9**. Furthermore, we found that when the 405 nm laser is constantly on, EB3-EGFP and tubulin-EGFP, despite being in different cells, show very similar intensity decrease profiles (**Figure S10**).

To better compare the difference of 405 nm laser interactions with ER and nuclei, we manually selected areas on and outside APX, as illustrated in **Figure S11**. When ER is treated, the tubulin-EGFP signal decrease pattern on and outside ER APXs are very similar. This further indicates that the intensity decrease is not induced by photobleaching but by cellular functional changes. Comparing tubulin-EGFP signal outside APXs for ER treatment and nuclei treatment, the latter shows a lesser signal decrease (**Figure S4c,d**). This suggests that 405 nm interacting with ER gives more pronounced cellular functional disruptions, likely induced by ROS.

8. Absorption and emission spectra of fluorescent dyes and laser/filter selections

The absorption and emission spectra of BODIPY 505/515 nm and the ER-Tracker Red are shown in **Figures S12a, b**. A schematic illustration of the laser wavelengths used for chemical selection, optical manipulation, and readout, together with the spectral windows of the two PMT channels are shown in **Figure S12c**. The chemical selection channel detects fluorescence signals from BODIPY, ER-Tracker, or mCherry-histone2B, while the readout channel detects fluorescence signals of EB3-EGFP or EGFP-tubulin.

9. Discussion on the EGFP signal decreases in different conditions

Considering the overall fluorescence signals of the whole cell, two factors can result in the EGFP signal decrease when conjugated with either EB3 or tubulin.

- Photobleaching by the 473 nm laser, which typically has a 36 μW power at the sample
- ROS generation induced by 405 nm laser, which has a power of either 150 or 240 μW

The 589 nm laser does not result in photobleaching of the EGFP signals. First, the 589 nm laser is outside of the excitation range of the EGFP. Second, the power of the 589 nm laser is 10 μW on the sample, which is much lower than the 473 nm and 405 nm lasers.

The 405 nm laser does not induce detectable photobleaching for EGFP. This statement is confirmed in **Figure S6**, in which 240 μW 405 nm laser is turned on only at the EB3 comets. The 405 nm laser interacts with only the EB3-EGFP molecules in real time in HeLa cells but does not induce a more significant EGFP signal loss compared to the control group (only with a 473 nm laser). This is because the EGFP has low absorption at the 405 nm wavelength. This statement is also confirmed in **Figure S7**.

The 473 nm laser (readout laser) at 36 μW scanning throughout the sample does induce a slow signal decrease of the EGFP, likely due to the photobleaching. This factor is measured in **Figures 2** and **4** as the control group to compare with ROS-induced EGFP signal loss. The ROS generated by 405 nm in different organelles increases the EGFP signal loss to different degrees, as quantified in **Figures 2** and **4**.

The activation of photoswitchable inhibitor PST-1 and the inhibition of microtubule polymerization do not change the overall EGFP signals in the whole cell, as discussed in **Supplementary Note 5** and **Figure S7**. This is because the EB3-EGFP or EGFP-tubulin molecules detached from microtubules due to inhibition are released into the cytoplasm, resulting in the loss of comet or fiber structures but an increase in the overall background. Therefore, when quantifying the EGFP signal of the entire cell, microtubule polymerization inhibition does not give a signal decrease. However, if only part of the cellular area is analyzed, as shown in **Figure 5b-d** and **Figure 6c-e**, the EGFP in the selected region (e.g. centrosome area) can decrease due to the redistribution of the EGFP conjugated EB3 or tubulin molecules. The areas with much higher microtubule polymerization such as the centrosome can result in a much higher signal decrease compared to other parts of the cell. On the contrary, the areas with low microtubule polymerization would show a temporary EGFP signal increase followed by a signal decrease that is induced by 473 nm laser photobleaching, as shown in **Figure 6e**. Note that when controlling the photoswitchable inhibitors, the 405 and 532 nm lasers are kept to a very low power range (< 30 μW) to avoid photobleaching or ROS generation.

Supplementary Figures

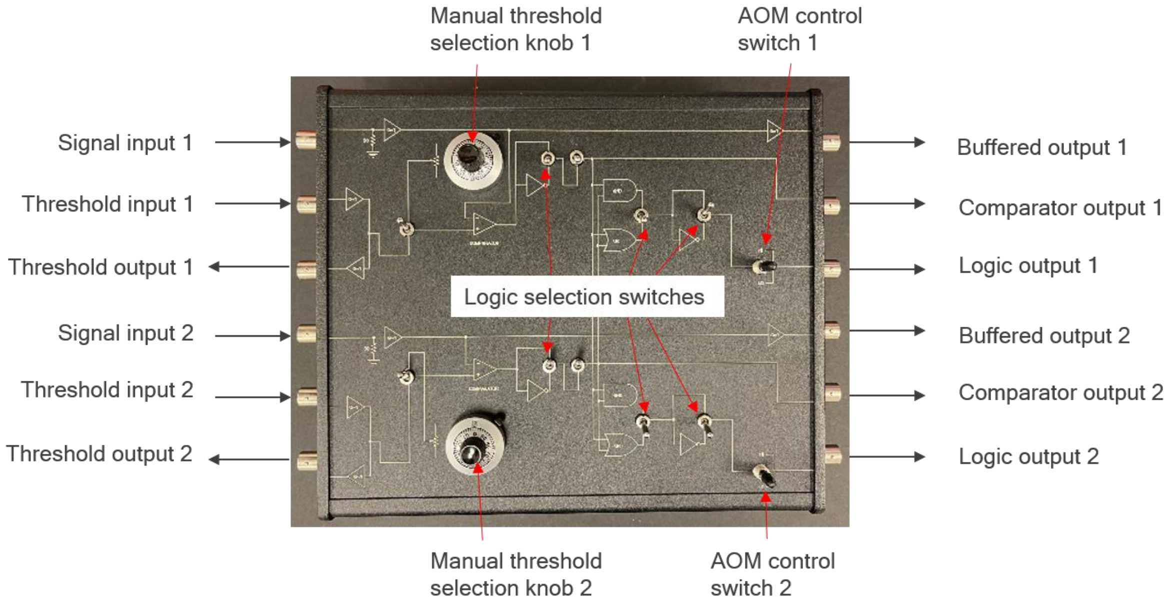

Figure S1. A photograph shows the design of the two-channel comparator circuit box. AOM: acousto-optic modulator.

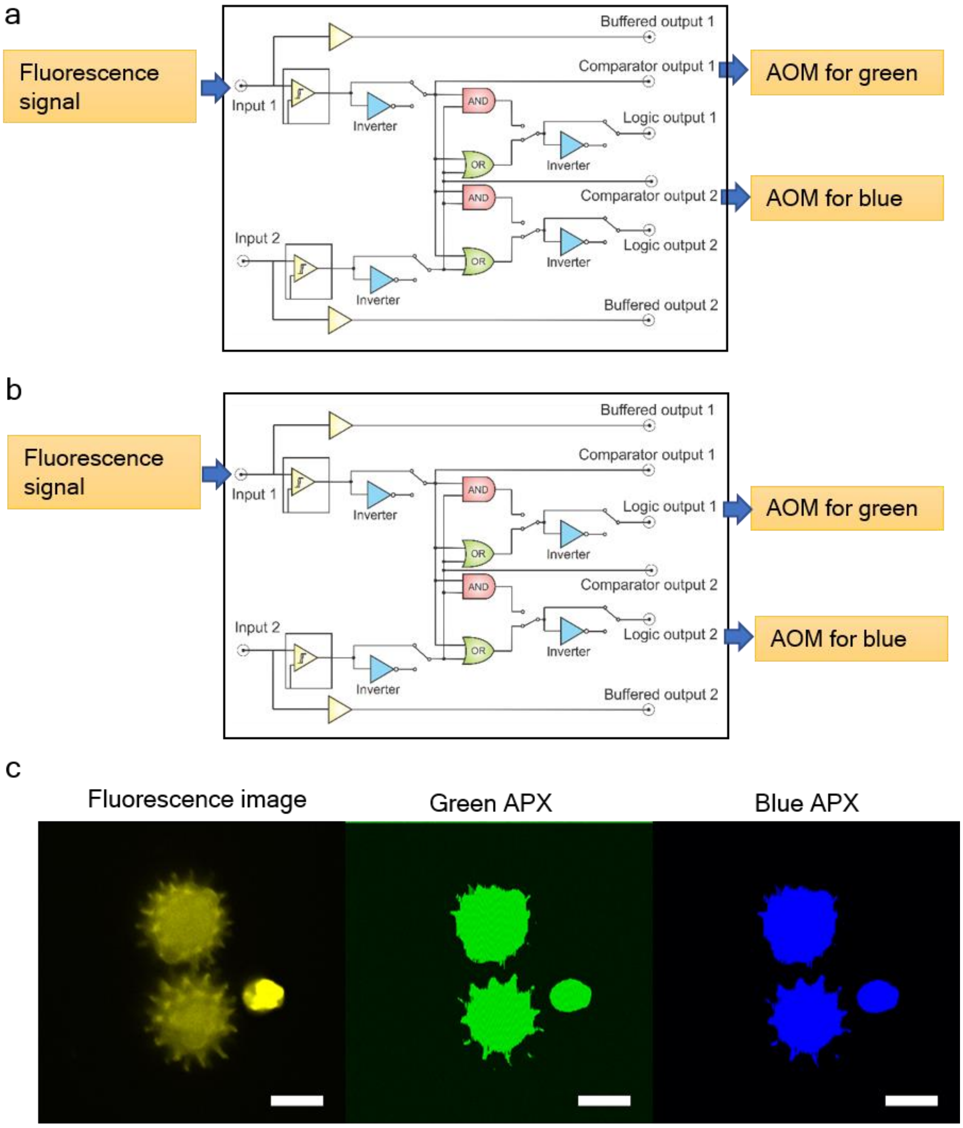

Figure S2. (a, b) Two ways of controlling blue (405 nm) and green (532 nm) lasers separately using comparator circuit boxes. The APXs for blue and green lasers are selected for the same chemical contrast simultaneously. (c) A fluorescence image from pollens and the corresponding APXs for green and blue lasers selected from the same signal. Scale bars are 20 μm.

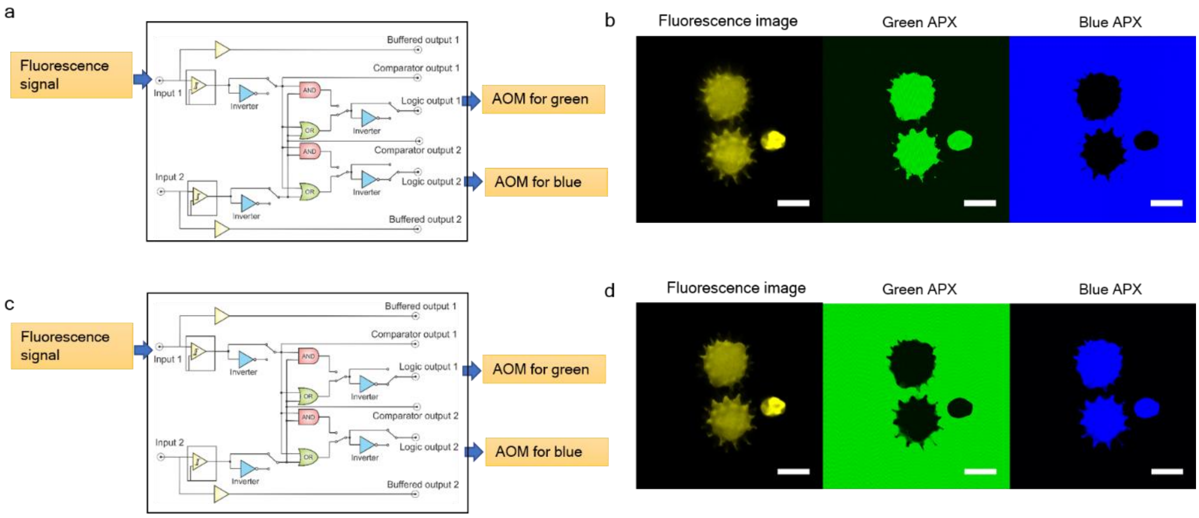

Figure S3. (a) An example comparator circuit connection for achieving compensating APXs for blue and green lasers. (b) A fluorescence image from pollens and the corresponding APXs for green and blue lasers. The APXs for the green laser are selected on the pollen, while the APXs for the blue laser are selected outside the pollen simultaneously. (c, d) Similar to panels (a) and (b), but invert APXs for blue and green. Scale bars are 20 μm.

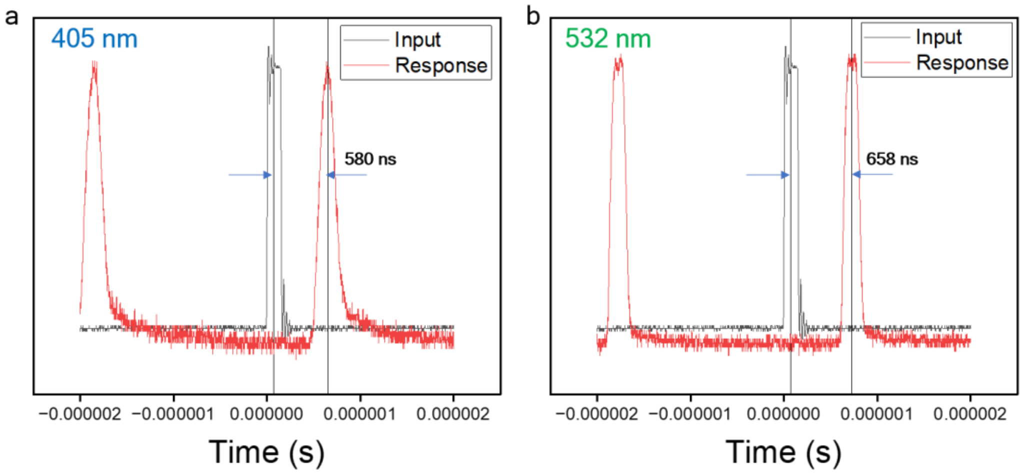

Figure S4. (a) The response time of the RPOC feedback system measured by a function generator and a photodiode for the 405 nm laser. (b) Similar to panel (a), but for the 532 nm laser.

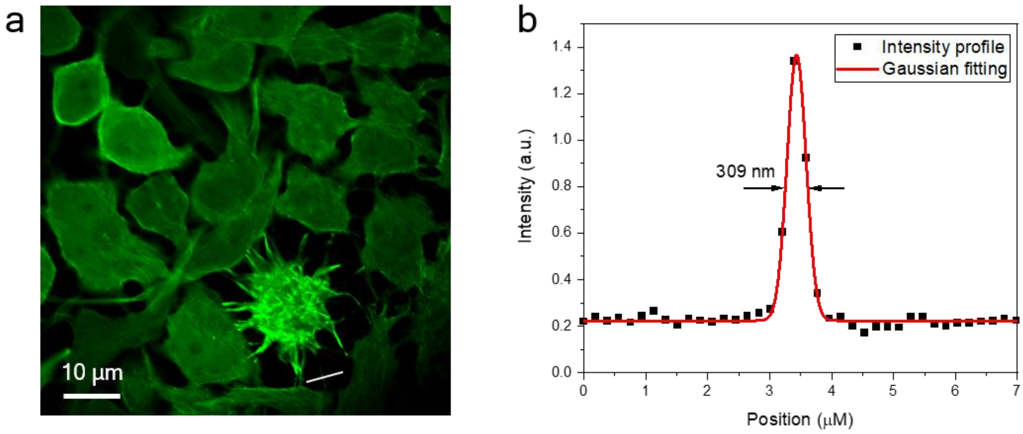

Figure S5. (a) A fluorescence image of EB3-EGFP signals from HeLa cells. The images are averaged for 10 frames at 10 microsecond pixel dwell time. Signals are excited by the 473 nm laser. (b) The intensity profile along the line in panel (a). Gaussian fitting gives a 309 nm spatial resolution.

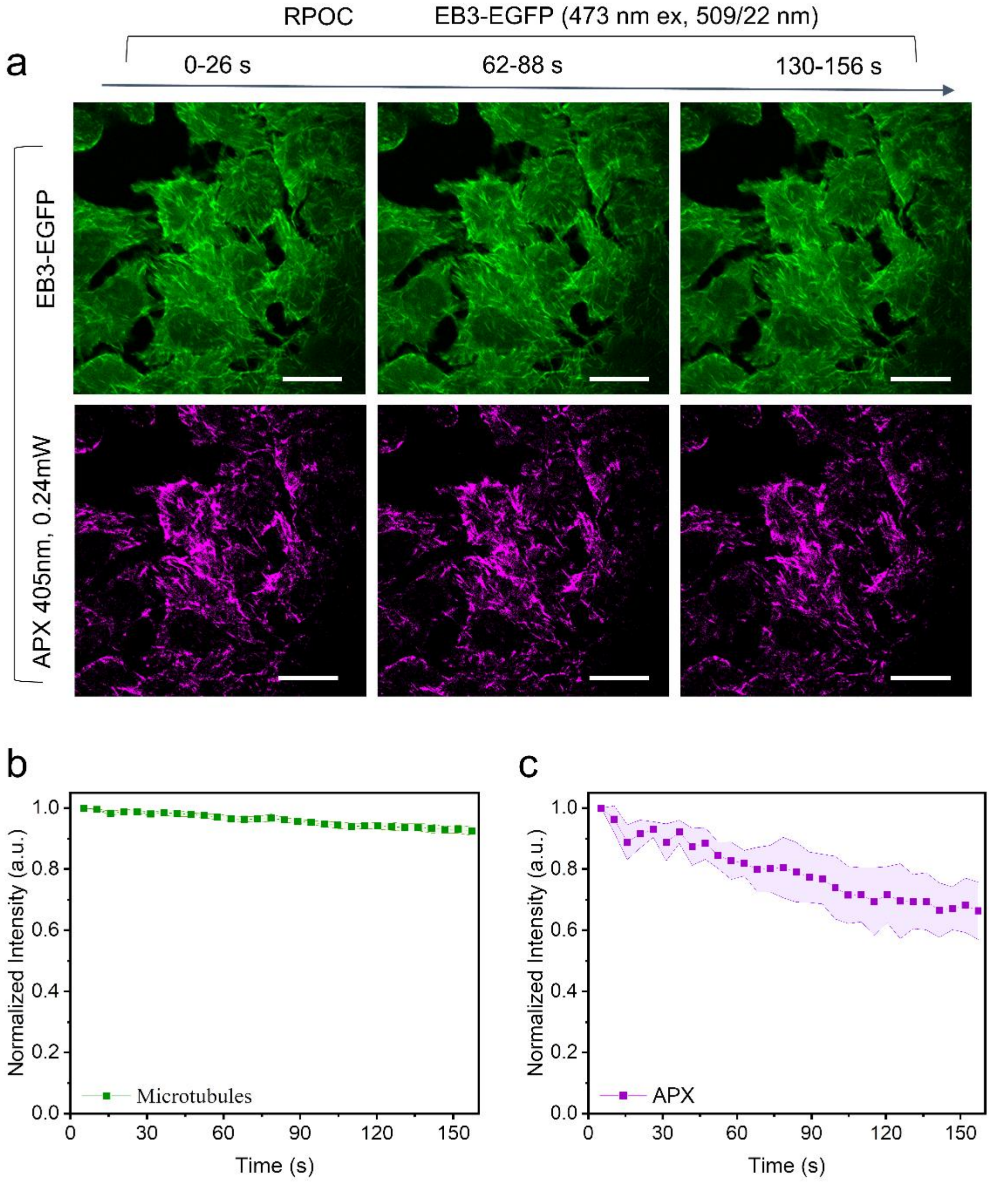

Figure S6. (a) EB3-EGFP signals from HeLa Kyoto EB3-EGFP cells and the corresponding APX signals which are selected by EB3-EGFP signals in different time windows during RPOC. (b) Time-dependent EB3-EGFP signal change during RPOC using APXs in panel (a) with 240 μW 405 nm laser. (c) The corresponding time-dependent APX signal change. Scale bars are 20 μm.

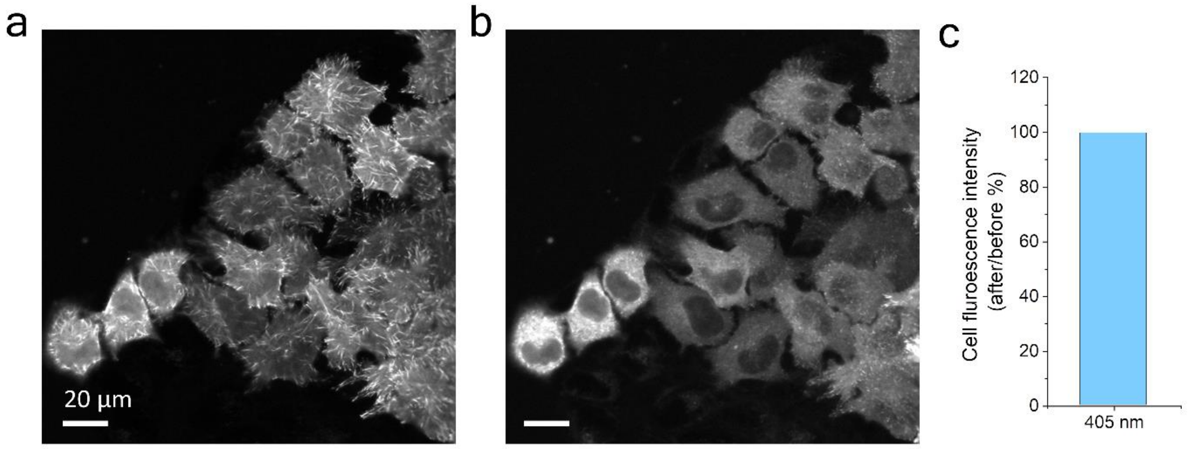

Figure S7. (a) A fluorescence image of EB3-EGFP signals from HeLa cells acquired using a commercial confocal fluorescence microscope. The excitation laser is 488 nm with 80 μW laser power. The pixel dwell time is 1.6 μs. (b) Image from the same field-of-view (FOV) after 160 μW 405 nm laser interaction for 10 minutes throughout the FOV. (c) The fluorescence intensity ratio (I_{after}/I_{before}) from the same FOV after and before laser treatment.

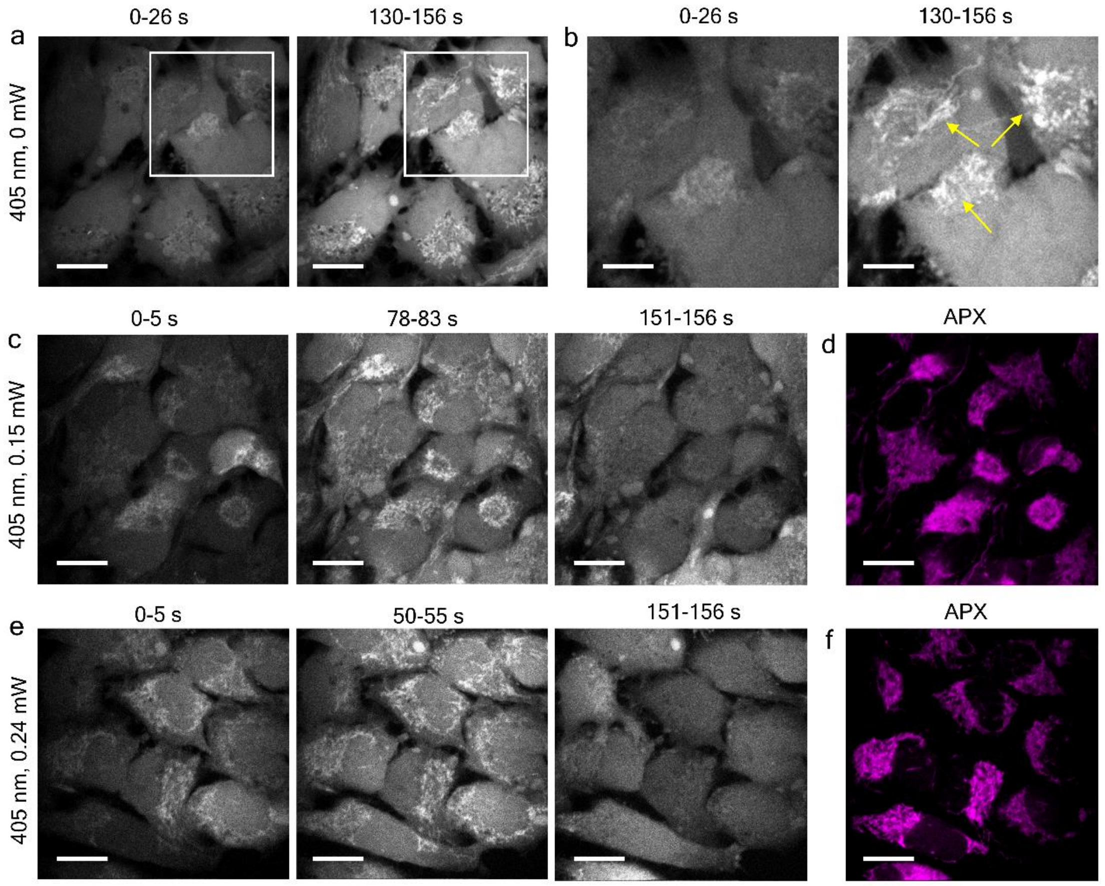

Figure S8. (a) Fluorescence intensity of H2DCFDA in HeLa cells averaged in different time windows. The cells are labeled using ER-Tracker Red and are illuminated by 22 μW 473 nm laser and 10 μW 589 nm laser constantly to visualize H2DCFDA and ER-Tracker. (b) Magnified areas in panel (a). Arrows point out mitochondria structures with stronger signals. (c) Fluorescence intensity of H2DCFDA in HeLa cells averaged in different time windows when ER is illuminated by 150 μW 405 nm laser. (d) APX on ER for panel (c). (e) Similar to panel (c) but treated ER with 240 μW 405 nm laser. (f) APX on ER for panel (e). Scale bars are 20 μm in panels (a), (c), (d), (e), and (f). Scale bars are 10 μm in panels (b).

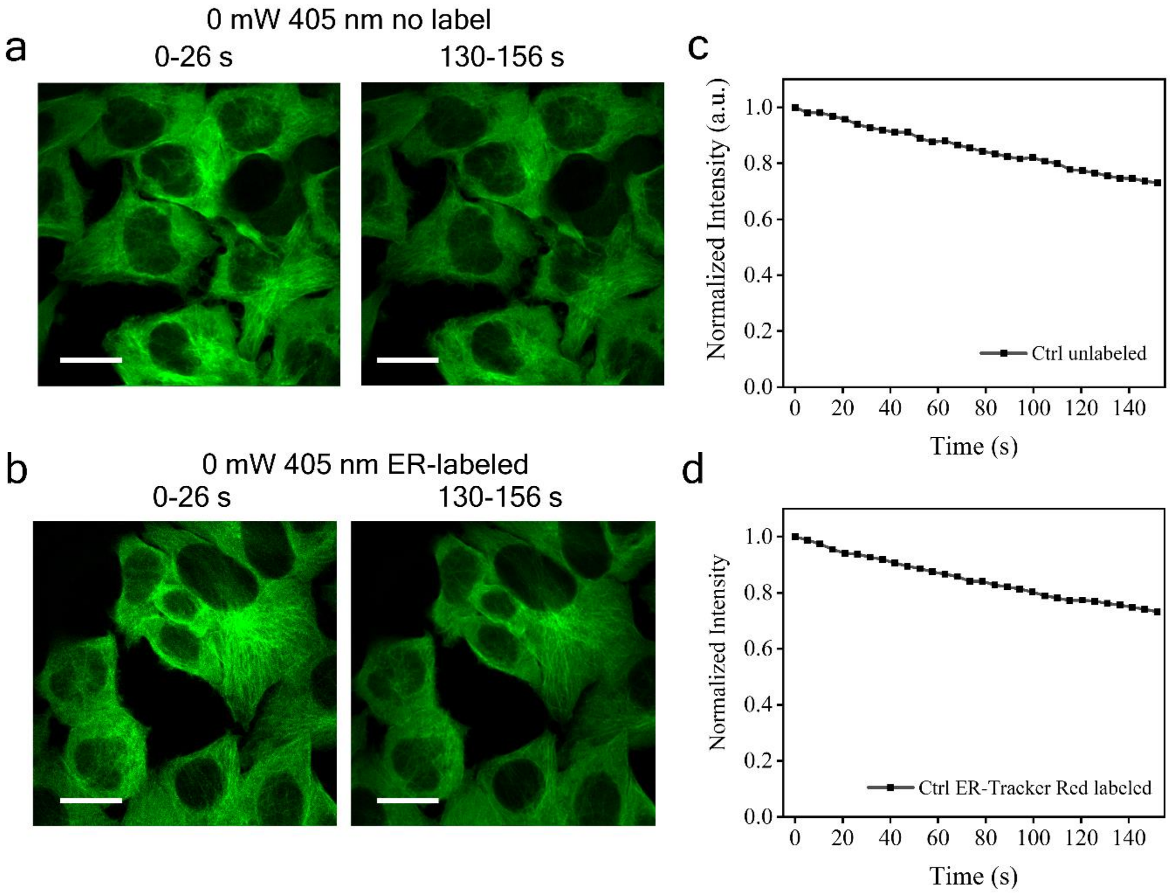

Figure S9. (a) EGFP-Tubulin signals from unlabeled HeLa Kyoto EGFP-alpha-tubulin/H2B-mCherry cells averaged in different time windows. Cells are illuminated by 36 μW 473 nm laser and 10 μW 589 nm laser constantly. (b) Similar to panel (a), but from the same cells labeled with ER-Tracker Red. (c) and (d) Tubulin-EGFP signal change as a function of time for panels (a) and (b), respectively. Scale bars are 20 μm.

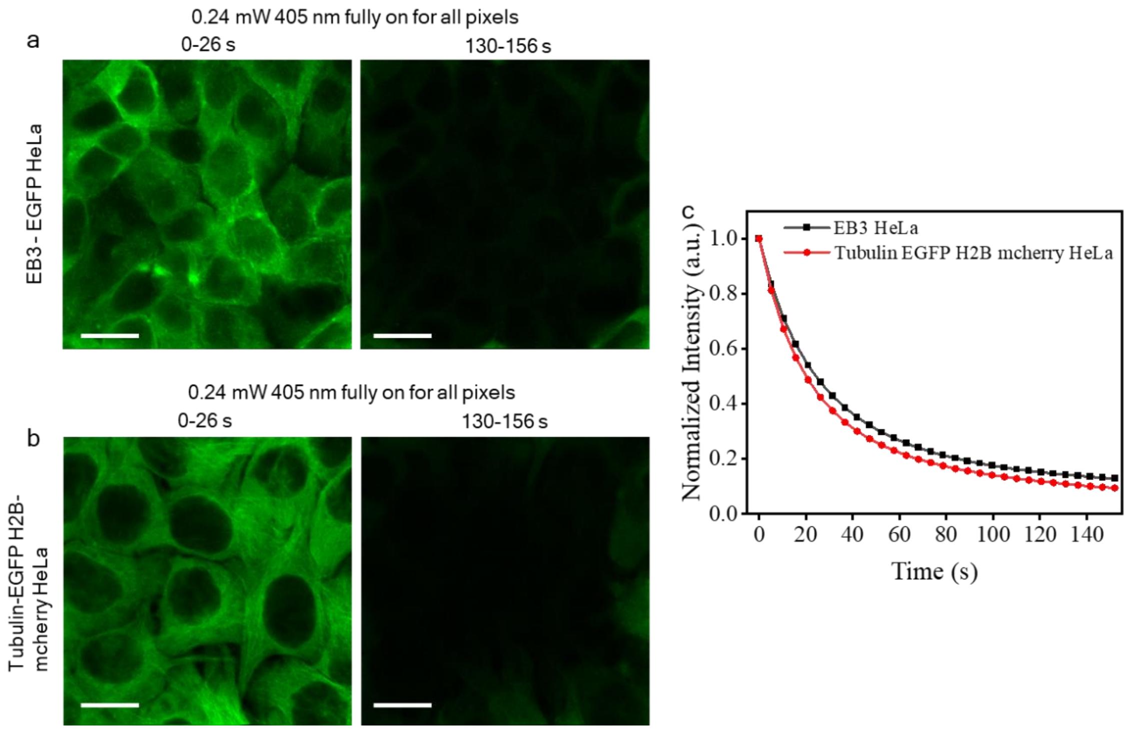

Figure S10. (a) EB3-EGFP signals from HeLa Kyoto EGFP-EB3 cells averaged in different time windows. The cells are illuminated by 36 μW 473 nm laser, 10 μW 589 nm laser, and 1.1 mW 405 nm laser constantly. (b) Similar to panel a but for EGFP-tubulin signals from HeLa Kyoto EGFP-alpha-tubulin/H2B-mCherry cells. (c) EB3-EGFP and EGFP-tubulin signal changes as a function of time for panels (a) and (b). Scale bars are 20 μm.

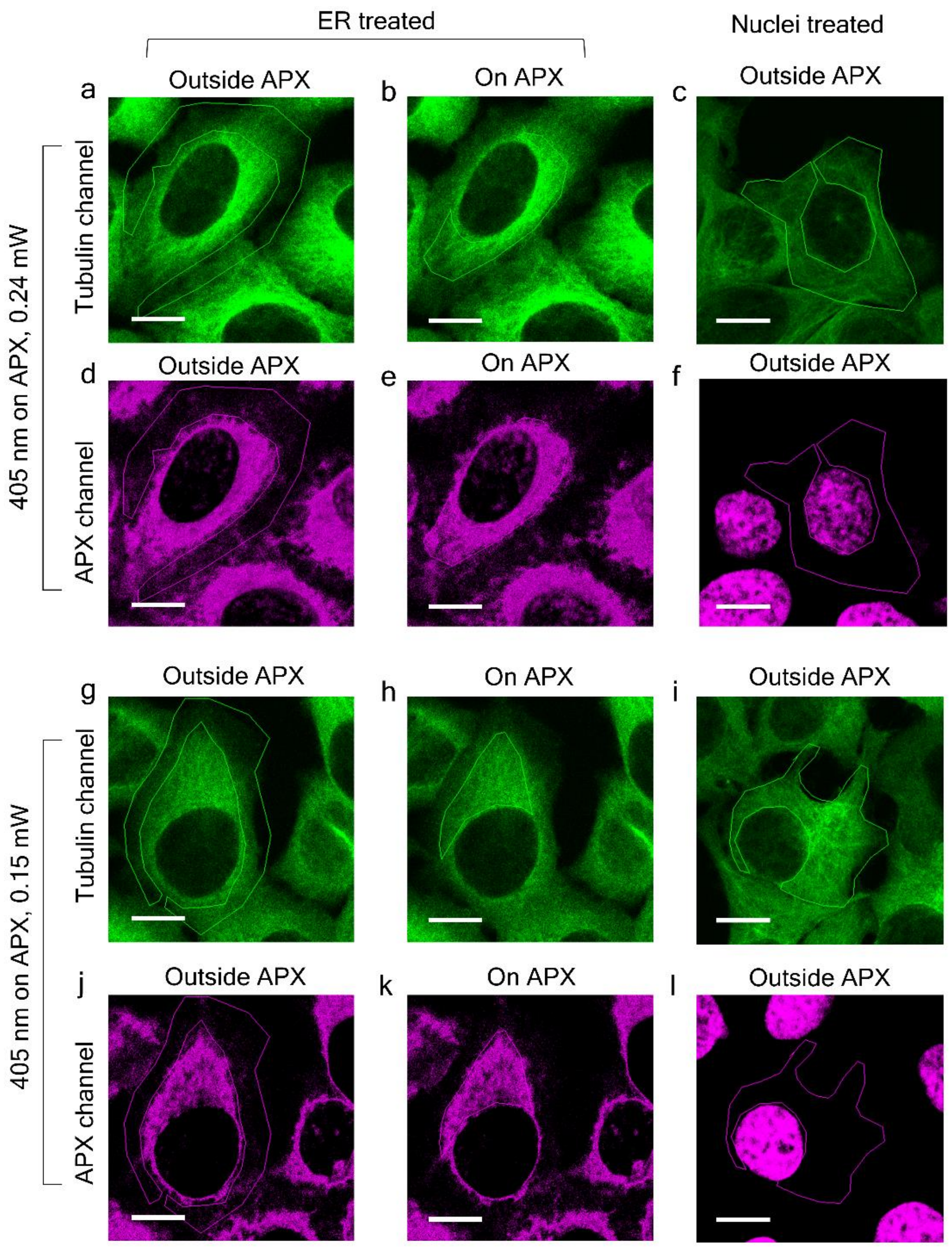

Figure S11. EGFP-tubulin signals and example areas selected to quantify the signal changes outside (a) and on (b) APXs. The cells are HeLa Kyoto EGFP-alpha-tubulin/H2B-mCherry cells labeled with ER-Tracker Red. APXs are selected on ER. (c) Similar to panels (a), but for unlabeled HeLa Kyoto EGFP-alpha-tubulin/H2B-mCherry cells. APXs are selected on nuclei. (d)-(f) The corresponding APX channel and the same selected areas for analysis. For panels (a)-(f), the cells are illuminated by 36 μW 473 nm laser and 10 μW 589 nm laser constantly. On APXs, 240 μW 405 nm laser is activated. (g)-(I) Similar to panels (a)-(f), but with 150 μW 405 nm laser on APXs. Scale bars are 10μm.

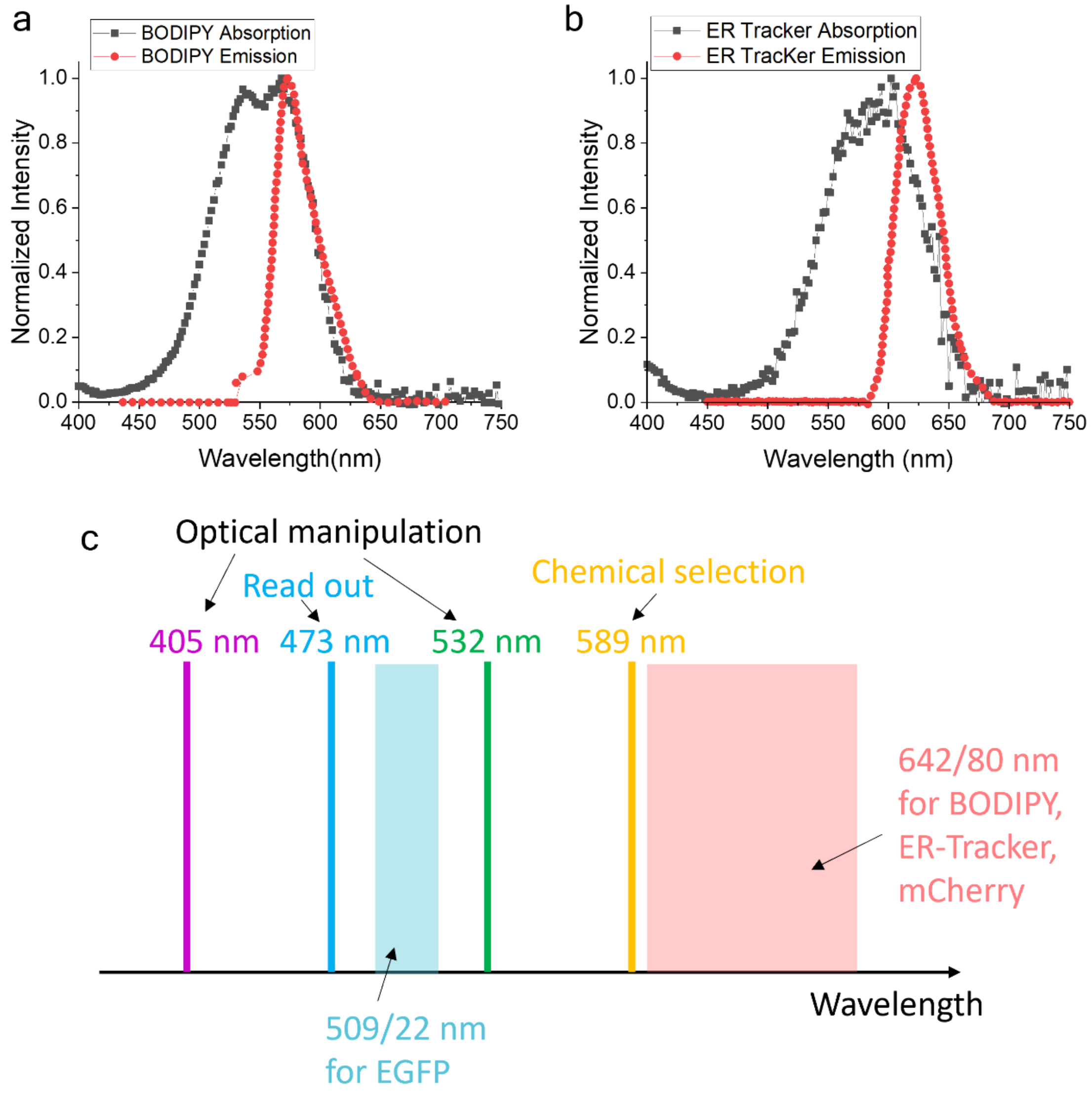

Figure S12. (a) The absorption and emission spectra of BODIPY 505/515 nm. (b) The absorption and emission spectra of ER-Tracker Red. (c) The laser wavelengths used for chemical selection, optical manipulation, and readout, and the filter passband windows for the two PMT channels. The 509/22 nm filter is for the readout channel, and the 642/80 nm filter is for the chemical selection channel.

Supplementary Movies
For all movie panels, the size of the FOV is 60 µm. Each panel consists of 30 frames with 512×512 pixels. The time interval between each frame is around 5.2 seconds. Movie S2 is combined by four panels, while Movies S3-S6 are combined by three panels.

Movie captions

Movie 1.1 Time-lapse EB3-EGFP signals in HeLa cells excited by 473 nm laser and detected by the CW-RPOC system.

Movie 1.2 Time-lapse APX intensity from 405 nm laser tracking high-intensity EB3 comets in HeLa cells.

Movie 1.3 Overlaid EB3-EGFP and APX time-lapse images of HeLa cells.

Movie 2. Time-lapse EB3-EGFP signals from four laser interaction conditions in **Figure 2**.

Movie 3. BODIPY signals from LDs, APX selected from BODIPY signals, and the corresponding EB3-EGFP signals from HeLa cells during RPOC. The cells are treated with inactivated PST-1 before RPOC.

Movie 4. ER-Tracker signals from ER, APX selected from ER-Tracker signals, and the corresponding EB3-EGFP signals from HeLa cells during RPOC. The cells are treated with inactivated PST-1 before RPOC.

Movies 5 and 6. APXs from blue and green lasers at different parts of HeLa cells and the corresponding EB3-EGFP signals from cells during RPOC. The cells are treated with inactivated PST-1 before RPOC.
